# Postoperative hyperglycemia among adult non-diabetic surgical patients at University of Gondar Comprehensive Specialized Hospital, Northwest Ethiopia

**DOI:** 10.1186/s12871-024-02592-9

**Published:** 2024-07-01

**Authors:** Eshetu Tesfaye Dejen, Misganaw Mengie Workie, Tadael Gudayu Zeleke, Biruk Adie Admass, Debas Yaregal Melesse, Tadesse Belayneh Melkie

**Affiliations:** 1 Department of Anesthesia, College of Medicine and Health Sciences, Bahar Dar University, Bahar Dar, Ethiopia; 2 Department of Anesthesia, College of Medicine and Health Sciences, University of Gondar, Gondar, Ethiopia

**Keywords:** *Blood glucose*, *Dysglycemia*, *Insulin resistance*, *Non-diabetic*, *Postanesthesia care unit*, .*Postoperative hyperglycemia*

## Abstract

**Background:**

Postoperative hyperglycemia is associated with morbidity and mortality in non-diabetic surgical patients. However, there is limited information on the extent and factors associated with postoperative hyperglycemia. This study assessed the magnitude and associated factors of postoperative hyperglycemia among non-diabetic adult patients who underwent elective surgery at University of Gondar Comprehensive Specialized Hospital, Northwest Ethiopia.

**Methods:**

A facility-based cross-sectional study was conducted among 412 adult patients who underwent elective surgery at University of Gondar Comprehensive Specialized Hospital from April 14 to June 30, 2022 All consecutive postoperative non-diabetic elective surgical patients who were admitted to PACU during the data collection period and who fulfilled inclusion criteria were included in the study until the intended minimum sample size was achieved. And data were collected through interviews using a pretested semi-structured questionnaire. Postoperative hyperglycemia was defined as a blood glucose level of ≥ 140 mg/dl. Multivariable logistic regression was performed to identify the association between postoperative hyperglycemia and independent variables. Variables with a p-value less than 0.05 and a 95% confidence interval (CI) were considered statistically significant.

**Results:**

A total of 405 patients’ data were evaluated with a response rate of 98.3%. The median (IQR) age was 40 (28-52) years. The prevalence of postoperative hyperglycemia was 34.1% (95% CI: 29.4–39.0). Factors significantly associated with postoperative hyperglycemia included being overweight (AOR = 5.45, 95% CI: 2.46-12.0), American Society of Anesthesiologists (ASA) classification II and III (AOR = 2.37, 95% CI: 1.17–4.79), postoperative low body temperature (AOR = 0.18, 95% CI: 0.069–0.48), blood loss ≥ 500 ml (AOR = 2.33, 95% CI: 1.27–4.27), long duration of surgery, mild pain (AOR = 5.17, 95% CI: 1.32–20.4), and moderate pain (AOR = 7.63, 95% CI: 1.811–32.20).

**Conclusion and recommendation:**

One-third of the study participants had postoperative hyperglycemia. Weight, ASA classification, postoperative body temperature, duration of surgery, intraoperative blood loss, and postoperative pain were identified as a modifiable risk factors. Maintaining normal body temperature throughout the procedure, treating postoperative pain, and monitoring and controlling blood glucose level in patients at risk of hyperglycemia is crucial.

## Introduction

According to the American College of Endocrinology and the American Association of Clinical Endocrinologists, hyperglycemia is defined as any blood sugar level exceeding 140 mg/dl (> 7.8 mmol/l) [1]. Stress hyperglycemia generally refers to transient hyperglycemia during illness and is usually restricted to patients without previous evidence of diabetes mllitus [2].

Surgical patients without a history of previous perioperative diabetes (DM) are prone to hyperglyciemia due to sympatatic stimulation associated with surgery, anesthesia, pain and trauma. Neurohormonal function is disrupted, as a result of which stress-induced release of counterregulatory hormones leads to hepatic gluconeogenesis and glycogenolysis [3–6]. It is also associated with acute insulin resistance and increases catecholamine and cortisol, leading to stress hyperglycemia [7].

High blood glucose levels are often seen in the postoperative period, affecting 40% [8] of non-cardiac surgeries and 60% of cardiac surgeries [9, 10]. Postoperative hyperglycemia is one modifiable risk factor for increased morbidity, increased hospital costs, and mortality in non-diabetic patients compared with diabetic patients [11–13].

It has been associated with postoperative complications in different types of surgical procedures such as general surgery [14], orthopedic surgery [15], spine surgery [16], head and neck surgery [17], hepatobiliary-pancreatic surgery [18], vascular surgery, and cardiac surgery [9, 19]. These complications include postoperative surgical site infection [20, 21], risk of pneumonia, systemic blood infections, urinary tract infections, skin infections, acute renal failure [13], and anastomotic leakage [22]. These complications can increase the risk of readmission, length of hospital stay and hospital costs in the postoperative period [23, 24]. Postoperative blood glucose (BG) ≥ 200 mg/dL was associated with worse outcomes than those patients experienced either euglycemic or mild postoperative hyperglycemia [25].

A mean perioperative glucose levels greater than 220 mg/dL were associated with a seven times higher risk of infection including wound infections, pneumonia, urinary tract infections, and sepsis in orthopedic trauma patients with no known history of diabetes mellitus [15].

Hyperglycemia has historically been considered beneficial and harmless in healthy adults without known diabetes [2, 26]. However, several studies have shown that hyperglycemia has been associated with poor clinical outcomes compared to those who maintain a euglycemic state [10, 25, 27–31]. Cross-sectional studies have shown that the risk of complications and mortality relates to the severity of hyperglycemia, with a higher risk observed in patients without a history of diabetes (new onset and stress-induced hyperglycemia) compared to those with a known diagnosis of diabetes [12, 13]. Evidence showed that non-diabetic patients with perioperative hyperglycemia had nearly twice the risk of infection, re-operative intervention, and in-hospital death than diabetic hyperglycemic patients [11]. Frisch et al. found an increased risk of 30-day mortality in nondiabetic patients with hyperglycemia when compared to patients with diabetes [13]. Hyperglycemia in non-diabetics has been also associated with impaired glucose intolerance or diabetes during follow-up testing [32, 33]. A study showed that among patients with admission hyperglycemia, 60% of them had confirmed diabetes at 1 year [2].

Despite the increased incidence and increased morbidity and mortality associated with hyperglycemia, perioperative blood glucose levels in nondiabetic surgical patients are often overlooked, undertreated, and unmonitored [27]. Study show that controlling hyperglycemia in the early postoperative period leads to faster recovery after major surgery, fewer complications, and lower hospital costs [34].

According to the literature, patient baseline characteristics affect the likelihood of postoperative hyperglycemia. Some of the recognized risk factors for the development of POH are black ethnicity, female sex, immunosuppression, higher BMI, comorbidity, extended stress response, low body temperature, and higher glycosylated hemoglobin (HgA1c) [35, 36].

Recognizing the limitations of knowledge and the need for knowledge to prevent the development of POH, assessing the problem and related factors is important. The primary objective of the study was to assess the prevalence and associated factors of POH Aamong non-diabetic adult surgical patients at the compherensive specialized Hospital of the University of Gondar in Northwest Ethiopia.

## Methods and materials

An institutional -based cross-sectional study was conducted to assess the prevalence and associated factors of postoperative hyperglycemia among adult non-diabetic surgical patients at the University of Gondar Comprehensive Specialized Hospital in Northwest Ethiopia from April 14 to June 30, 2022. The study included all adult non-diabetic patients who underwent elective surgery at the University of Gondar Comprehensive Specialized Hospital and were admitted to the Post-Anesthesia Care Unit (PACU). General surgery, orthopedic surgery, gynecology surgery, and neurosurgery were included in this study. Ethical approval was obtained from the institutional review board (IRB) of the University of Gondar, School of Medicine, with reference number SoM/14/02/2022. Informed consent was obtained from each participant after being informed of the details of the study.

### Inclusion and exclusion

Inclusion criteria encompassed non-diabetic patients aged 18 years and older who underwent elective surgery and were admitted to the PACU. Exclusion criteria comprised patients with previously diagnosed diabetes or a known HgA1c > 6.5%, individuals with known or suspected pregnancy, patients taking oral hypoglycemic agents or insulin, and those undergoing surgeries lasting less than 30 min.

### Sample size determination and sampling technique

The sample size was determined using the single population proportion formula with the assumptions of the proportion of postoperative hyperglycemia (42%) among adult aged ≥ 18 years in Addis Ababa, Ethiopia [37] 95% Confidence interval(CI) (z = 1.96), 5% margin of error, and 10% non-response rate for the compensation of the potential non-responses. The final sample size was 412.

A probability sampling method was used. All consecutive postoperative non-diabetic elective surgical patients who were admitted to PACU during the data collection period and who fulfilled inclusion criteria were included in the study until the intended minimum sample size was achieved.

### Study variables

Postoperative hyperglycemia was the dependent outcome variable. While age, sex, weight, BMI, presence of comorbidity, ASA status, preoperative blood glucose level, hypoproteinemia, preoperative hemoglobin level, family history of DM, type of anesthesia, use of perioperative steroids, amount of intraoperative IV fluid, type of procedure, duration of surgery, postoperative pain, postoperative body temperature, amount of intraoperative blood loss, and blood transfusion were the independent variables.

### Operational definition

#### Hyperglycemia

Defined as an elevated fasting blood sugar level of ≥ 140 mg/dL (7.8 mmol/L) [1],whereas severe hyperglycemia was defined as a blood glucose level ≥ 180 mg/dl [1, 38].

### Data collection procedures and tools

A semi-structured questionnaire was prepared by the principal investigator (ETD) and other research team members. Questions in the questionnaire include sociodemographic data, preoperative (ASA status, preoperative hemoglobin, history of medical illness such as hypertension, cardiovascular disease, and history of medication usage such as antihypertensive, beta-blockers, and steroids), intraoperative (types of surgical procedures, amount of intraoperative fluid given, amount of intraoperative blood loss, intraoperative transfusion, intraoperative opioid given, intraoperative vasopressor use, and duration of surgery), and postoperative (postoperative pain and postoperative body temperature) variables. Sociodemographic and preoperative clinical data were extracted from the patient’s records on the morning of surgery. Intraoperative anesthetic and surgical data were collected from the intraoperative anesthesia charts. The surgical service was made aware of all glucose levels and was free to treat hyperglycemia(BG > 140 mg/dl) or hypoglycemia (BG < 70 mg/dl). Blood glucose was re-measured if CBG was > 200 mg/dl postoperatively. The management of abnormal glucose levels was the decision of the surgical service (surgeon, anesthetist, and nurse).

### Measurements

Blood glucose level was measured in the PACU by a trained anesthetist in PACU and recorded on the patient chart. The glucometer used for capillary blood glucose measurement was a CareSens N Eco blood glucose meter with a 100% accuracy of ± 15 mg/dl in a range of 20-600 mg/dl. The glucose meters were calibrated as per the manufacturer’s recommendations. The glucometer was provided by the primary investigator. Hyperglycemia was considered when the blood glucose level was ≥ 140 mg/dl and if the blood glucose level was less than 70 mg/dl was considered hypoglycemia. The assigned data collectors measured and records the BG level from the PACU follow-up chart within 30 min of the patient’s arrival at PACU till patient discharged from the PACU (usually around two hours after surgery).

The temperature of the participants was measured after the patient arrived in the PACU using a digital axillary thermometer (digital thermometer type, code DI777 with an accuracy of +/- 0.1 ºc in the range of 32–42 ºc by placing the probe of the thermometer in the armpit close to the axillary artery while tightly adducting the arm.

Pain also assessed using the Numeric Rating Scale (NRS) 60 min after the patient arrived in the PACU. Pain-in a numeric rating scale (NRS) was described as 0 -no pain, 1–3 Mild pain, 4–6 moderate pain, and 7–10 severe pain [39].

Body Mass Index (BMI) was calculated based on the weight in kilograms (kg) and height in square meters (m²). The BMI categories were defined as follows: underweight (< 18.5 kg/m²), normal (18.5 to 24.9 kg/m²), overweight (25 to 29.9 kg/m²), and obese (> 30 kg/m²) [40].

### Data quality control measures and management

A one-day training session was provided to the data collectors and supervisors, covering the study’s objectives, data collection procedures, and how to maintain the confidentiality and privacy of the study subjects. Prior to the actual data collection, a pre-test was conducted at the University of Gondar Comprehensive Specialized Hospital two weeks in advance to assess the questionnaire’s completeness, fulfillment and understandability. A questionnaire was developed by reviewing various sources from existing literature. To ensure its validity, a pretest was conducted with a sample representing 5% of the target population. Importantly, these individuals were not included in the main study. Based on the feedback and results from this pretest, necessary modifications were made to the questionnaire to enhance its clarity, relevance, and overall effectiveness.

### Data management and statistical analysis

Data were coded, edited, entered, and cleaned in Epidata version 4.6 before being exported to the Stata/SE statistical package (ver.14, StataCorp, College Station, TX) for analysis. Descriptive statistics like frequencies, percentage, mean with standard deviation (SD), median and inter-quartile range (IQR) was calculated. Normality of data was checked using the Shapiro-Wilk test and expressed accordingly. The results were presented by using text, tables, charts, and graphs.

Prevalence of POH was calculated as a proportion of patients with postoperative hyperglycemia and was reported with 95% Confidence Interval (CI). Bivariate and multivariate logistic regression analyses were done to determine the presence of associations between dependent and independent variables, and the odds ratio with 95% CIs was used to determine the degree of association between dependent and independent variables. All variables with a p-value < 0.2 in the bivariate logistic regression were entered into the multivariate model. Model fitness was checked by Hosmer–Lemeshow goodness of fit test. P values < 0.05 were considered significant.

## Results

A total of 412 patient data sets were collected in the PACU. After evaluating the data, seven patients were excluded due to incomplete information, resulting in a response rate of 98.3%. Consequently, 405 patients were included in the analysis, comprising 86 orthopedic surgeries, 286 general surgeries, 30 gynecologic surgeries, and 3 neurosurgeries.

### Sociodemographic and perioperative clinical characteristics of study participants

The median age of the patients was 40.0 years (Interquartile Range [IQR] 28–52 years). The majority (40.4%) of the patient’s age was found in the 18–40 age group, followed by the age group 41–60 (32.6%), and the rest are above 60 years. Majority of the paraticipants were male (50.4%). More than three-quarters of the participants had normal BMIs (78%). Regarding American Society of Anesthesiologists (ASA) status, most of the patients were under ASA class I (65.9%), the rest were under class II and III. Most of the participants underwent general surgery (70.6%) with general anesthesia (55.3%) which took a median (IQR) duration of 150 (120–180) minutes. Mild pain was reported in 49.6% of the participants. Table 1 shows the socio-demographic and perioperative clinical characteristics of the study participants.

**Table 1:** Socio-demographic and perioperative clinical characteristics of postoperative surgical patients at UOGCSH Northwest Ethiopia, April 14-June 30, 2022(*N* = 405)

Variable	Postoperative Hyperglycemia		Frequency *N*(%)
	Yes *N*(%)	No *N*(%)	
Sex			
Female	92 (45.8)	109(54.2)	201(49.6)
Male	46(22.5)	158(77.5)	204(50.4)
BMI category(mean ± SD)	22.17 ± 2.6	21.43 ± 2.1	
Underweight	8 (27.6)	21(72.4)	29(7.2)
Normal weight	95(30.1)	221(69,9)	316(78)
Overweight	35(58.3)	25(41.7)	60(14.8)
Hypoalbuminemia			
No	43(53.5)	37(46.5)	80(19.8)
Yes	24(57.1)	18(42.9)	42(10.4)
Missing	71(25.1)	212(74.9)	283(69.8)
Anemia			
No	121(33)	246(67)	367(90.6)
Yes	17(44.7)	21(55.3)	38(9.4)
ASA classification			
ASA I	62(23.2)	205(76.8)	267(65.9)
ASA II and III	76(55.1)	62(44.9)	138(34.1)
Type of anesthesia			
Regional anesthesia	33(18.2)	148(81.8)	181(44.7)
General anesthesia	105(46.9)	119(53.1)	224(55.3)
Type of surgery			
Orthopedic surgery	8(9.3)	78(90.7)	86(21.2)
General surgery	106(37.1)	180(62.9)	286(70.6)
Gynecologic surgery	22(73.3)	8(26.7)	30(7.4)
Neurosurgery	2(66.7)	1(33.3)	3(0.8)
Blood loss (ml)			
< 500 ml	48(19.5)	198(80.5)	246(60.7)
≥ 500 ml	90(56.6)	69(43.4)	159(39.3)
Postoperative pain			
No	3(5.5)	52(94.5)	55(13.6)
Mild	60(29.9)	141(70.1)	201(49.6)
Moderate	75(50.3)	74(49.7)	149(36.8)
Duration of surgery in minute(median, IQR)*	170	150	150(120–180)
Postoperative body temperature (median, IQR)*	36.25	36.4	36.4(36.1–36.5)

NB:- BMI-Body Mass Index, SD = Standard Deviation, IQR-Inter Quartile Range, continuous variables (*) are expressed as median (IQR)

### Prevalence of postoperative hyperglycemia

Of the total 405 participants, 138 had postoperative hyperglycemia, resulting in an overall prevalence of POH of 34.1% (95% CI: 29.4–39.0) in the early postoperative period. Out of those, 38(9%) had developed severe postoperative hyperglycemia.

### Factors associated with postoperative hyperglycemia

Following the univariate analysis, the factors shown to have a significant effect on POH were; age, gender, preoperative beta-blocker use, preoperative fasting hour (Nil per Os (NPO)), and type of anesthesia. In multivariate analysis, after controlling potential confounding factors, BMI ≥ 25, ASA physical status II and III, type of surgery, duration of surgery, intraoperative blood loss, postoperative body temperature, and postoperative pain were significantly associated with POH. Accordingly, the odds of having postoperative hyperglycemia were five times higher in the patient group who have BMI ≥ 25 kg/m2 compared to participants with normal BMI (AOR = 5.45, 95% CI:2.46,12.06). Being ASA ≥ II had 2.33 times morelikelly to to develop POH compared to their counterparts (AOR = 2.37, 95% CI:1.27–4.27). Similarly, those participants with intraoperative blood loss of more than 500 ml demonstrated 2.33 times more likely at risk of developing POH compared to participants with blood loss < 500 ml (AOR = 2.33, 95% CI:1.27–4.27). However, the odds of POH decreased by 82% as body temperature increased by 1ºc (AOR = 0.18, 95% CI: 0.069–0.48) (Table 2).

**Table 2:** Bivariable and multivariable logistic regression analysis of factors associated with postoperative hyperglycemia at UOGCSH NorthWest Ethiopia, from April 14 to June 30, 2022(*N* = 405)

Variable	Postoperative hyperglycemia		COR (95% CI)	AOR (95%CI)	*P*_value
	Yes *N*(%)	No *N*(%)			
Age			1.01(1.005- 1.03)*	1.009(0.99–1.03)	0.34
Gender					
Female	92 (45.8)	109(54.2)	1	1	
Male	46(22.5)	158(77.5)	0.33(0.22–0.53)**	0.85(0.42–1.72)	0.66
BMI category					
Underweight	8 (27.6)	21(72.4)	0.88(0.37–2.07)	0.63(0.22–1.75)	0.37
Normal weight	95(30.1)	221(69,9)	1	1	
Overweight	35(58.3)	25(41.7)	3.25(1.84–5.84)**	5.45 (2.46–12.1)	0.000
Beta-blocker					
No	107(31.3)	231(67.7)	1	1	
Yes	31(46.3)	36(53.7)	1.85(1.09–3.16)*	0.66(0.29–1.49)	0.32
ASA classification					
ASA I	62(23.2)	205(76.8)	1	1	
ASA II and III	76(55.1)	62(44.9)	4.0(2.61–6.29)**	2.37(1.17–4.79)	0.016
Type of anesthesia					
Regional anesthesia	33(18.2)	148(81.8)	1	1	
General anesthesia	105(46.9)	119(53.1)	0.25(0.15–0.40)**	1.17(0.48–2.82)	0.72
Type of surgery					
Orthopedic surgery	8(9.3)	78(90.7)	1	1	
General surgery	106(37.1)	180(62.9)	5.4 (2.66–12.35)**	4.08(1.39–11.99)	0.010
Gynecologic surgery	22(73.3)	8(26.7)	26.8(9.03–79.60)**	14.0(3.3-60.27)	0.000
Neurosurgery	2(66.7)	1(33.3)	19.6(1.58–239.5)	9.21(0.59–142)	0.112
Blood loss (ml)					
< 500 ml	48(19.5)	198(80.5)	1	1	
≥ 500 ml	90(56.6)	69(43.4)	5.38(3.44–8.39)**	2.33(1.27–4.27)	0.006
Postoperative pain					
No	3(5.5)	52(94.5)	1	1	
Mild	60(29.9)	141(70.1)	7.37(2.21–24.54)**	5.17(1.32–20.4)	0.018
Moderate	75(50.3)	74(49.7)	17.56(5.25–58.75)**	7.63(1.811–32.2)	0.006
Duration surgery (minute)***	170	150	1.01(1.006–1.014)**	1.006(1.00-1.01)	0.028
Postoperative body TºC***	36.25	36.4	0.16(0.076–0.36)**	0.183(0.06 -0.48)	0.001

NB: - COR = Crude odds ratio, CI confidence interval, Tº= temperature in degree Celcius

1 = refference group *Significant at < 0.05 **Significant at < 0.001 for bivariable logistic regression. ***=continous variable(median), Hosmer lemon show model fitness(*p* = 0.98)

## Discussion

In our study, the prevalence of POH was 34.1% and BMI greater and equal to 25 kg/m2, ASA physical status II and III, type of surgery, intraoperative blood loss more than 500 ml, postoperative low body temperature, having postoperative pain, and long duration of surgery were found to be associated with POH. In this section, the major findings of the current study are discussed in light of previous empirical evidence from the literature.

The prevalence of POH found in this study 34.1% (95% CI: 29.4–39),which is almost in line with other studies conducted in Asia(34.28%) [41], Europe (33.3%) [42], and the USA(39%) [9]. However, it is lower than the prevalence reported in studies conducted in the USA(53%) [43], Italy (55%) [25], Singapore (56.1%) [10], and Ethiopia (42%) [37]. The possible reasons for these discrepancies might include differences in the cutoff points for determining hyperglycemia, the timing of blood glucose measurements, the nature of the surgical procedures, the types of study populations, and socio-demographic and clinical differences between Ethiopia and other countries. Conversely, the findings of our study are higher than those of studies conducted in the USA (25.2%) [38], Brazil (26.4%) [44], and Canada(20%) [45]. These variations might be due to differences in study settings, the use of higher cutoff points for hyperglycemia, and the timing of blood glucose measurements. Importantly, one possible reason for the relatively higher prevalence of POH in our study is that 99% of the participants were premedicated with dexamethasone to prevent postoperative nausea and vomiting. A single dose of dexamethasone has been identified as a risk factor for hyperglycemia because it can stimulate gluconeogenesis and inhibit peripheral insulin action [44]. Therefore, blood glucose concentration should be monitored in nondiabetic patients undergoing surgery who receive dexamethasone, and clinicians should use their judgment before administering this medication.

The prevalence of POH in this study is clinically important since hyperglycemia is associated with poor clinical outcomes in nondiabetic surgical patients. This finding indicates that hyperglycemia is common in the postoperative period, underscoring the importance of vigilant monitoring and controlling abnormal blood glucose levels.

BMI was found to be associated with POH in the current study. Overweight participants (BMI ≥ 25 kg/m²) (AOR = 5.45, 95% CI = 2.46–12.06) were about five times more likely to develop hyperglycemia compared to those with a normal BMI. Our result is higher than that of a study conducted in Egypt [46]. However, it is supported by studies from Brazil [44], and studies from the USA [47, 48]. The possible justification for this result may be increasing BMI is associated significantly with insulin resistance [49]. Higher levels of BMI are associated with increases in glucose and mid-blood pressure as well as triglyceride levels [50]. Contrary to our findings from a study in Finland, BMI was not associated with the occurrence of hyperglycemia [42]. This can be due to the smaller sample size in their subgroup. Our finding showed that patients with high BMI have a risk of POH, and hence, their blood glucose should be monitored during the postoperative period.

Our result also revealed that a respondent with ASA’s physical status II and III was three times greater likely to develop hyperglycemia compared to participants with ASA I (AOR = 2.37 95% CI = 1.27–4.27). Critically ill patients may have altered glucose metabolism due to insulin resistance, which may explain this increase in blood glucose. Our result is supported by a study from the USA [48]. Only ASA I, II, and a small number of ASA III patients have participated in this study. The absence of patients with higher ASA classes during the study period may have affected our result. Those with higher ASA classification need an emphasis on blood glucose monitoring and achieving euglycemia seems beneficial to these patients.

A study showed that severe pain reduced insulin sensitivity by 22%, which affects glucose metabolism. It is conceivable that the counterregulatory hormonal response plays an important role [51]. However, epidural analgesia as an adjunct to general anesthesia results in lower levels of BG and insulin needed postoperatively in patients undergoing surgery [52]. This study demonstrated that participants who had mild and moderate pain were 5 times and 7 times more likely to develop POH compared to the participants who had no pain (AOR = 5.17, 95% CI = 1.32–20.4) and (AOR = 7.63, 95% CI = 1.81–32.20) respectively. However, the small sample size in these subgroups should be noted. Based on our results, pain relief in stressful situations is important for reducing the surgical stress response and maintaining normal glucose metabolism. Whenever possible, better pain control using multimodal analgesia may enhance insulin action.

Unlike previous studies, this study demonstrated that low body temperature is associated with postoperative hyperglycemia. The odds of POH occurrence decreased by 82% as body temperature increased by 1ºc(AOR = 0.18, 95% CI = 0.069–0.48). Although we did not find research directly supporting this finding, scientific literature suggests that hypothermia may indirectly increase glucose levels in the bloodstream, resulting in hyperglycemia. Hypothermia initiates increased sympathetic activity, leading to elevated levels of catecholamines and free fatty acids, which in turn decreases insulin secretion and increases tissue resistance to insulin [53]. The increase in catecholamine release is compounded by impaired peripheral glucose uptake at the tissue level because of hypothermia. The insulin decrease is also caused by the cooling of the islets of Langerhans, responsible for insulin secretion [54, 55]. Maintaining normal body temperature will be crucial in the perioperative period.

A study demonstrates that increased blood loss is associated with hepatic insulin resistance [56]. Research from the USA [43] found higher postoperative blood glucose levels of patients with blood loss ≥ 500–999 ml were 15.4% higher than those with a blood loss of < 500 ml. Our study indicates the odds of POH were 2.33 times with Blood loss ≥ 500 ml than with < 500 ml blood loss. This finding is supported by Mohan et al., who observed that as blood loss increases in colorectal surgical patients, the risk of postoperative hyperglycemia also increases. Increased bleeding leads to hypovolemia and hypotension, activating the sympathetic adrenal system and the hypothalamic-pituitary-adrenal axis, resulting in metabolic changes such as hyperglycemia [57]. So, vigilant postoperative BG monitoring is critical for all patients who have a risk of increased blood loss.

The type of surgery was identified as a predictor of POH. We observed patients who underwent gynecologic and general surgery have higher odds of developing hyperglycemia compared to orthopedic surgical patients AOR = 14.0(95% CI (3.3-60.27)) and AOR = 4.08(95% CI (1.39–11.99). In support of this result, evidence suggests the severity of hyperglycemia depends on the type of anesthesia and the type of surgery, with higher glucose levels observed in general anesthetics or thoracic/abdominal surgery compared with epidural/local anesthesia or peripheral/laparoscopic surgery [58]. Since surgeries involving the abdomen and thorax have been correlated with a more prolonged and pronounced degree of hyperglycemia. Furthermore, less invasive procedures have been related to less increase in insulin resistance [59]. In contrast to our study, only neurosurgery was associated with the occurrence of postoperative hyperglycemia in the case of a study in Ethiopia [37]. The possible reason for this discrepancy may be due to the small number of neurosurgical patients in our study. The discrepancy may indeed be attributed to the small number of neurosurgical patients in our study. However, it’s important to note that while this difference may be statistically significant, it may not necessarily be clinically significant. The small number of patients in this subgroup could limit the generalizability of the findings and their relevance to clinical practice. Therefore, caution should be exercised when interpreting the results, and further research with larger sample sizes may be needed to determine the clinical significance of these findings.

Even though the majority of patients who had POH received general anesthesia (76%) there was no association between type of anesthesia and POH. The length and duration of the surgical intervention determine a great variation in the contribution of counterregulatory hormones this may be due to the sympathetic response associated with surgical stress and the release of counterregulatory hormones, which determine lower insulin secretion and peripheral tissue resistance to the action of insulin and produce hyperglycemia [60]. Our result showed that as the duration of surgery increased by 1 min, the occurrence of POH increased by 0.6%. This is consistent with studies from Brazil [44] and China [41]. Thus, our result revealed that the type of surgery and longer duration of surgery are associated with the occurrence of POH, which needs emphasis on monitoring BG and controlling the higher blood glucose level in the postoperative period.

### Limitations of the study

The limitations of this study include the fact that HbA1c measurements were not routinely performed in our setup. Consequently, the interpretation of results should be approached cautiously, as this was an exploratory study, and the variables included may serve as surrogates for others. Further research is necessary to determine the independent predictive value of each variable. Additionally, our study did not explore the association between postoperative hyperglycemia and postoperative clinical outcomes (complications). Moreover, controlling for confounding factors is particularly challenging in a cross-sectional study. Confounding factors, which can distort the apparent relationship between the studied variables, are difficult to manage without longitudinal data or more complex study designs.

Another significant limitation of our study is the small sample size. A larger sample would provide more robust data, allowing for a more accurate analysis and potentially more definitive conclusions.

## Conclusion

In conclusion, this study found a high prevalence of POH. Among adult non-diabetic surgical patients at PACU. Higher BMI, ASA physical status II and III, types of surgical procedures, increased intraoperative blood loss, postoperative pain, low body temperature, and longer duration of surgery were strongly associated with POH. These findings underscore the importance of vigilant monitoring and control of blood glucose levels in the postoperative period, particularly in patients with identified risk factors. Implementing strategies for better pain management, maintaining normal body temperature, and closely monitoring patients undergoing surgeries associated with higher risk of POH could potentially mitigate the occurrence of hyperglycemia and improve clinical outcomes in non-diabetic surgical patients.

### Recommendation

For healthcare professionals: Ensure maintenance of normal body temperature and effective management of postoperative pain. Implement regular blood glucose monitoring in the PACU, particularly for high-risk patients such as those with elevated body mass index and those who underwent invasive surgical procedures. Prioritize the control of severe hyperglycemia to mitigate associated risks and complications. For researchers conduct further studies to explore the implications of postoperative hyperglycemia on the development of postoperative complications. Investigate potential interventions to prevent or manage hyperglycemia in the perioperative period effectively.

By implementing these recommendations, healthcare providers can enhance patient care and outcomes while researchers can contribute to advancing knowledge and developing strategies to address postoperative hyperglycemia and its consequences effectively.

## Data Availability

data is provided within the manuscript.
